# Mechanistic Analysis of Physicochemical Cues in Promoting Human Pluripotent Stem Cell Self-Renewal and Metabolism

**DOI:** 10.3390/ijms19113459

**Published:** 2018-11-04

**Authors:** Nan Hai, Dong Woo Shin, Huanjing Bi, Kaiming Ye, Sha Jin

**Affiliations:** Department of Biomedical Engineering, Thomas J. Watson School of Engineering and Applied Sciences, State University of New York in Binghamton, Binghamton, NY 13902, USA; nhai1@binghamton.edu (N.H.); dshin26@binghamton.edu (D.W.S.); hbi1@binghamton.edu (H.B.); kye@binghamton.edu (K.Y.)

**Keywords:** human pluripotent stem cells, porous membrane, metabolism, polyethylene terephthalate, proliferation, apoptosis

## Abstract

We have previously reported that a porous membrane of polyethylene terephthalate (PET) enables significant augmentation of human pluripotent stem cell (hPSC) proliferation and differentiation. The interaction between hPSCs and the PET surface induces β-catenin-mediated wingless/integrated (*Wnt*) signaling, leading to upregulation of the expression of adhesion molecules in hPSCs. In this study, we sought to unveil mechanisms underlying the role of the PET membrane in hPSC self-renewal and metabolism. We discovered that physicochemical cues of the PET membrane considerably alter hPSC metabolism by increasing the cell yield and suppressing the generation of toxic byproduct, indicating an effective cell self-renewal and a less apoptotic culture environment in the membrane culture system. Furthermore, we discovered that a caspase-8 medicated apoptotic pathway plays a profound role in obstructing hPSCs grown on a traditional tissue culture plate (TCP). Treating hPSCs seeded on a TCP surface with a caspase-8 inhibitor significantly suppressed cellular apoptotic pathway and improved cell proliferation and metabolism. Our experimental results provided valuable insights into signal pathways influencing hPSC self-renewal during routine maintenance and expansion, which would shed light on large-scale preparation of hPSCs for clinical applications.

## 1. Introduction

Human pluripotent stem cells (hPSCs) are promising for clinical applications. Studies over the past decades have demonstrated that hPSC fate, including self-renewal, differentiation, and death, is determined decidedly by culture microenvironments, which are defined as niches throughout cell-extracellular matrix (ECM) or cell-cell interactions [1,2,3,4,5,6,7,8]. These niches include signal molecules [9,10], synthetic and nature substrates [5,11,12,13,14,15,16,17,18,19], and culture architectures (2D or 3D) [20,21,22,23]. These niches are unique and divergent from cultures of non-pluripotent stem cells. Particularly, hPSCs require specific microenvironments to support their attachment, spreading, and self-renewal. Adhesion molecule signaling plays an indispensable role for these cell events, which usually influences hPSCs through cell-molecule or cell-substrate interactions. Such interaction may include soluble and insoluble physicochemical factors.

Mechanistically, hPSC surface receptors, such as integrins, play various vital roles in cell interaction with ECM [4,5,18,24,25]. Insoluble mechanical factors may also control hPSC behavior, such as their morphology, attachment, spreading, proliferation, migration, and differentiation. These factors include, but are not limited to: Mechanical stress, topography of a substrate surface (e.g., roughness), and porous structure [26]. In our previous work, we reported that a polyethylene terephthalate (PET) porous membrane with 2 × 10^6^ pores/cm^2^ augments significantly human embryonic stem cell (hESC) proliferation and differentiation [4]. The study demonstrated that the interaction of cell-PET membrane stimulates a β-catenin-mediated *Wnt* signaling pathway, leading to enhanced hPSC attachment and proliferation.

In this study, we sought to investigate the mechanism underlying the enhancement of hPSC self-renewal by the PET membrane. We discovered that physicochemical cues provided by the porous membrane alter hPSC metabolism considerably, as indicated by a significant increase in cell yield and decrease in the production of toxic byproduct. Our experimental results revealed that the caspase-8 medicated apoptotic signaling pathway is suppressed in hPSCs grown on the PET membrane as compared to those on a traditional tissue culture plate (TCP). These observations suggested the obstruction of cell self-renewal on a TCP surface. Treating hPSCs grown on a TCP surface with a caspase-8 inhibitor yielded a cell proliferation rate comparable to that achieved on a PET membrane surface. These experimental results provided instructive insights into signal pathways influencing hPSC metabolism during routine maintenance and expansion, which would shed light on large-scale preparation of hPSCs for clinical applications.

## 2. Results

### 2.1. The Effect of Substrate Cues on hPSC Proliferation and Metabolism

**Figure 1:**
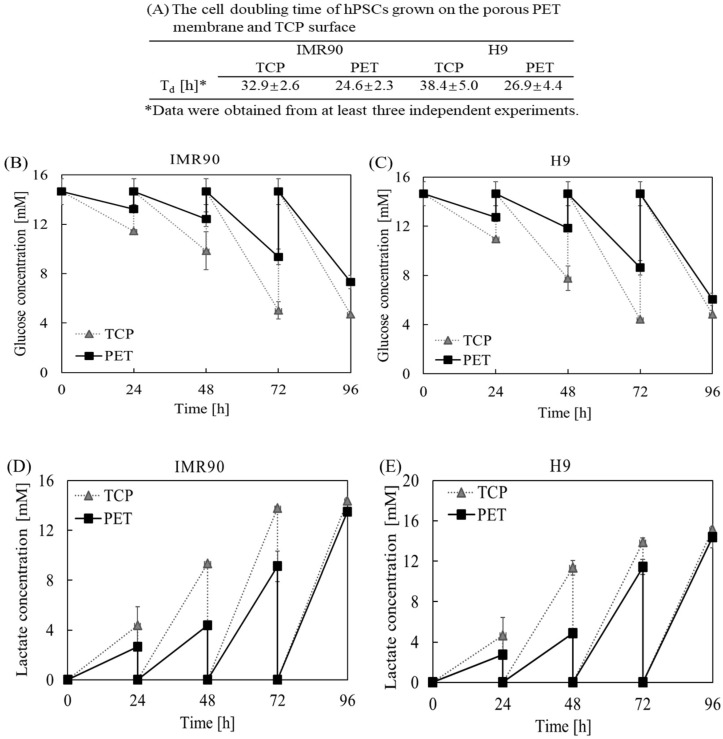
The proliferation and metabolism of human pluripotent stem cells (hPSCs) grown on the polyethylene terephthalate (PET) membrane and the tissue culture plate (TCP) surface. (**A**) Cell doubling times. The time course of glucose concentration (**B**,**C**) and lactate concentration (**D**,**E**) in induced pluripotent stem cell (iPSC) and human embryonic stem cell (hESC) cultures on the PET and TCP surfaces. Data shown were averages from at least three independent experiments.

**Figure 2:**
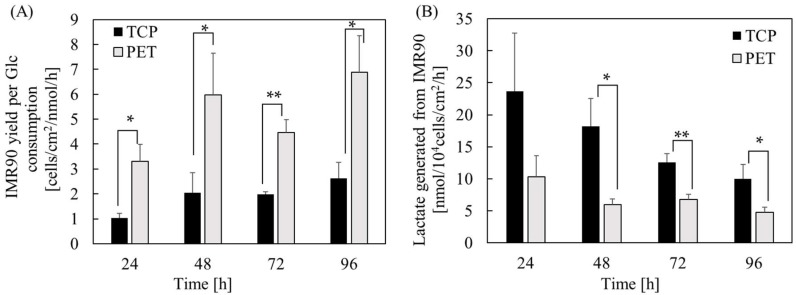

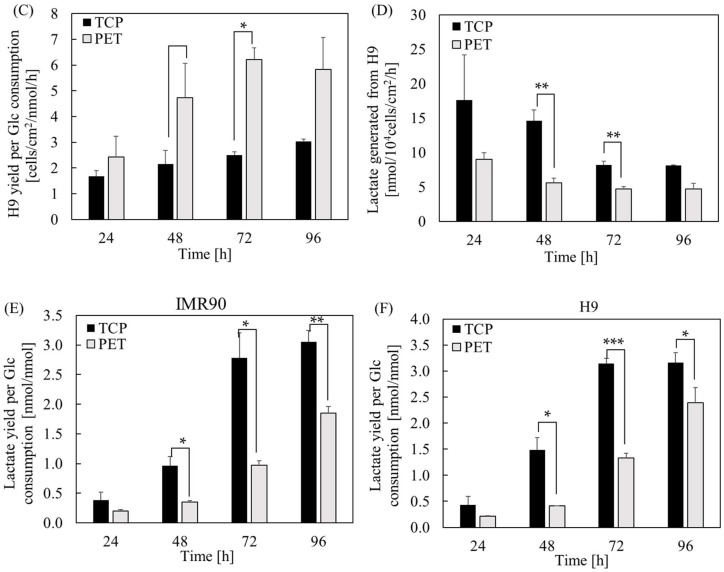
The time course of growth and metabolism of hPSCs grown on the PET membrane and TCP surface. (**A**,**C**) Cell yield based on glucose (Glc) consumption. (**B**,**D**) The amount of lactate generated per glucose consumed. (**E**,**F**) The ratio of lactate accumulated to glucose consumed. * *p* < 0.05; ** *p* < 0.01; *** *p* < 0.001.

To characterize the effect of PET substrate cues on hPSC proliferation and metabolism, we determined cell doubling time, glucose consumption, and lactate generation in induced pluripotent stem cell (iPSC) and human embryonic stem cell (hESC) cultures on the PET membrane and TCP surfaces. As shown in Figure 1, we observed a significant shorter cell doubling time in hPSCs grown on the PET membrane surface. The doubling time of IMR90 cells grown on the porous PET membrane was shortened to 24.6 ± 2.3 h, as compared to 32.9 ± 2.6 h when cultured on the TCP surface. Similarly, H9 cells cultured on the porous PET membrane and the TCP surface had a doubling time of 26.9 ± 4.4 h and 38.4 ± 5.0 h, respectively. Furthermore, we observed that cells grown on the TCP surface not only consumed more glucose, but also produced more byproducts, such as lactate (Figure 1B–E). The calculation of cell yield based on glucose consumption indicated that hPSCs had a much higher yield on the PET membrane surface (Figure 2A,C). The lactate is a byproduct produced from a cell when it metabolizes glucose through glycolysis. Not only is lactate an indication of inefficient adenosine triphosphate (ATP) production, it also has harmful effects on a cell. Lactate generated from a cell increases both osmolarity and acidity of the medium, which exerts an inhibitory effect on the metabolism of a cell. Interestingly, we found that the yields of lactate generated from hPSCs decreased significantly (Figure 2B,D). By comparing yields of lactate per glucose consumed, the PET membrane demonstrated superior performance in inhibiting lactate production during cell proliferation and metabolism, in comparison to the TCP surface (Figure 2E,F). These experimental results further divulged that the substrate cues of PET membrane play a superficial role in hPSC expansion.

### 2.2. Signal Pathways Governing the Enhancement of hPSC Proliferation and Metabolism

**Figure 3:**
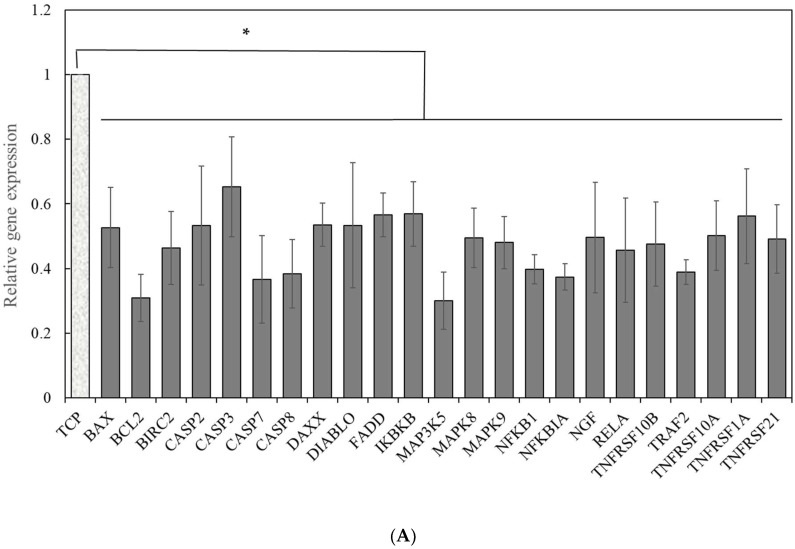

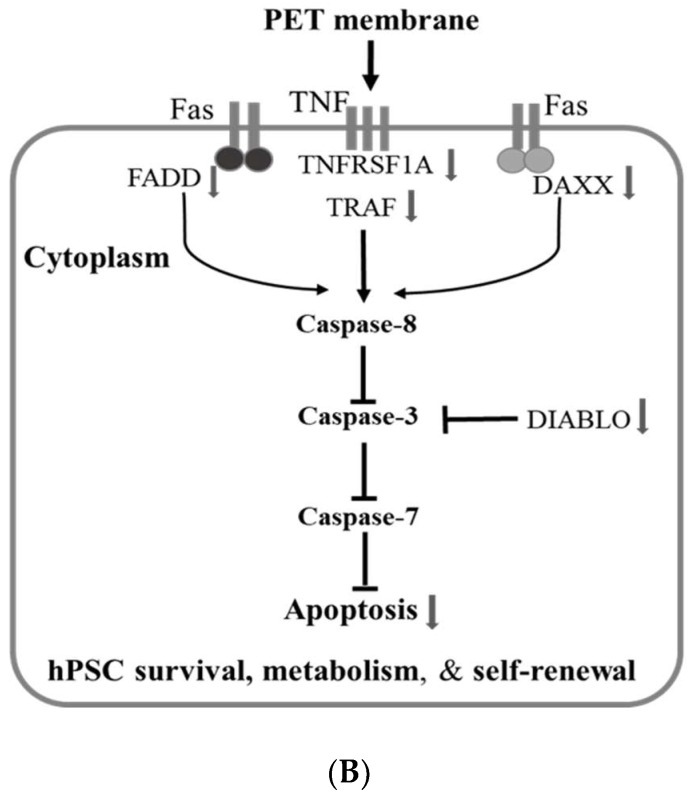
Mechanistic analysis of apoptotic signaling pathways in iPSCs grown on the PET membrane or TCP surface. (**A**) Comparison of apoptotic gene expressions in iPSCs grown on the PET membrane surface to those expressed in cells grown on TCP surface. The gene expressions in cells grown on the PET membrane surface were normalized to the levels on TCP. * *p* < 0.05. Data were calculated from three independent experiments. (**B**) The suppression of caspase-mediated apoptotic pathway by the PET membrane. Black arrows stand for signaling directions. Grey arrows denote gene downregulation. 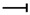 signifies inhibition.

We hypothesized that insoluble mechanical factors of PET may influence hPSC self-renewal. To understand the mechanisms underlying the enhancement of cell proliferation and metabolism by cell-PET membrane interaction, we characterized the expression of genes involved in JAK-STAT, apoptotic, and shear stress and mechanotransduction pathways. There were no significant differences in genes pertaining to JAK-STAT or shear stress and mechanotransduction pathways between PET and TCP condition. However, we discovered that a panel of genes associated with an apoptotic pathway was downregulated in cells grown on a PET membrane surface (Figure 3A). Among them, four are tumor necrosis factor (TNF) receptors, including tumor necrosis factor receptor superfamily member 1A (TNFRSF1A), tumor necrosis factor receptor superfamily member 10A (TNFRSF10A), tumor necrosis factor receptor superfamily member 10B (TNFRSF10B), and tumor necrosis factor receptor superfamily member 21 (TNFRSF21). It seemed that the TNF receptor-mediated apoptosis signaling pathway was suppressed in cells cultured on the PET membrane surface. Another four genes are caspases family genes, including Caspase-2 (CASP2), caspase-3 (CASP3), caspase-7 (CASP7), and caspase-8 (CASP8). These factors are crucial to apoptotic signaling pathways. 

Other related mediators, such as Fas-associated protein with death domain (FADD), diablo IAP-binding mitochondrial protein (DIABLO), and baculoviral IAP repeat-containing protein 2 (BIRC2), were also found to be downregulated in cells cultured on the PET membrane surface. Furthermore, as observed from Figure 3A, mitogen-activated protein kinase 5 (MAP3K5), mitogen-activated protein kinase 8 (MAPK8), mitogen-activated protein kinase 9 (MAPK9), and death domain associated protein (DAXX) in cells grown on the PET surface were two-fold down-regulated as compared to those on the TCP surface. The inhibitory expressions of the mitogen-activated protein (MAP) kinase family on the PET surface is a result of the reduced expression of the TNF receptor family as discussed above. DAXX and FADD are considered two distinct apoptotic pathways downstream of Fas [27]. These experimental results suggested that FADD, DAXX, and TNF-associated apoptotic pathways are suppressed in cells grown on a PET membrane surface (Figure 3B).

Captivatingly, inflammatory gene expressions of nuclear factor-κB (NFKB), nuclear factor-κB subunit 1 (NFKB1), inhibitor of nuclear factor κB kinase subunit β (IKBKB), and RELA were more than 2-fold downregulated as well (Figure 3A). Nuclear factor-κB is a transcription regulator that is activated by various intracellular and extracellular stimuli that induce inflammatory reactions. It leads to inappropriate immune cell development or delayed cell growth. On the other hand, the RELA gene encodes nuclear factor NF-κB p65 subunit protein, also known as p65. It is directly involved in NF-κB activation and modulates immune response [28]. The downregulation of these inflammatory genes suggested that nuclear factor- κB signaling is not a leading cell survival signal in the PET membrane surface culture. 

In addition, BAX, BCL2, and nerve growth factor (NGF) showed a 2-fold decrease in their gene expression levels on the PET, as compared to those on TCP. NGF can drive the gene expression of BCL2, stimulating cell proliferation and survival. Since the intrinsic apoptotic pathway is mostly mediated by the Bcl-2 family [29], it appears that the BCL2 signaling is dispensable in the PET membrane culture. These data suggested that the extrinsic pathway, rather than the intrinsic pathway, plays a key role in suppressing cell death and promoting cell spreading and proliferation in the PET membrane cultures (Figure 3B). These results expanded our understanding on the mechanism underlying the enhancement of cell self-renewal on the PET membrane surface. Accordingly, we speculated that a porous PET membrane sends extrinsic signals to cells through the cell–PET membrane interaction. The decreased expressions of CASP-3, -7, and -8 in cells cultured on the PET membrane surface suggested declining of the apoptotic pathways, leading to a shortened cell doubling time and efficient metabolism (Figure 3B). All these results divulged collectively that cells cultured on a TCP surface experience caspase-mediated apoptosis. 

### 2.3. The Effect of Inhibition of Caspase-8-Mediated Pathway on the Self-Renewal of hPSCs Grown on the TCP Surface

**Figure 4:**
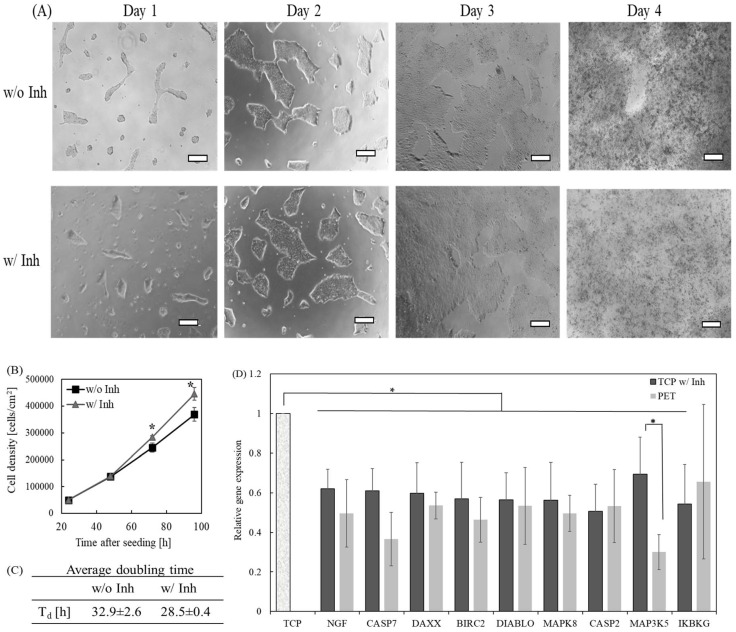
Caspase-mediated apoptotic pathway plays a crucial role in cells grown on the TCP surface. (**A**) Morphology of iPSCs cultured on the TCP surface without and with treatment of caspase-8 inhibitor (Inh). Scale bar: 250 μm. (**B**) The time course of cell proliferation on the TCP surface without and with treatment of caspase-8 inhibitor (Inh). (**C**) Average cell doubling time on the TCP surface without and with caspas-8 inhibitor treatment. (**D**) Comparison of gene expressions in cells cultured on the TCP surface after treating with caspase-8 inhibitor and the PET membrane surface. Gene expressions were normalized to those expressed on the TCP surface without inhibitor treatment. * *p* < 0.05. Data were calculated from three independent experiments.

According to the above experimental results and data interpretation, it seems like caspase-8 is the principal component governing hPSC proliferation and metabolism observed in Figure 1, Figure 2 and Figure 3 (Figure 3B). To validate whether the caspase-mediated apoptosis that the cells experience on a TCP surface can be alleviated, we used a caspase inhibitor to suppress cellular apoptotic signaling pathways during cell culture. First, we treated iPSCs with a caspase-8 inhibitor, Z-IETD-FMK, before seeding the cells on a TCP surface. We found that the cells could not attach to the TCP surface, suggesting the inhibition of cell attachment by Z-IETD-FMK. Next, we added Z-IETD-FMK (50 μM) to the cell culture medium at 24 h after seeding. The caspase-8 inhibitor was removed from the cell culture medium after two hours of incubation. The cells were cultured for an additional three days after the removal of the inhibitor. Remarkably, we observed a significant improvement of cell proliferation after treatment with caspase-8 inhibitor (Figure 4A,B). The cell doubling time was shortened to 28.5 ± 0.4 h after caspase-8 inhibitor treatment (Figure 4C), similar to those grown on the PET membrane surface. We carefully monitored cell morphology daily. There were no morphological changes from cells cultured with and without caspase-8 treatment. The cells showed typical, undifferentiated morphology throughout the entire period of cell culture. 

To examine further apoptotic status in cells grown on the TCP surface after inhibitor treatment, we compared expressions of apoptotic genes in these cells to those detected on the PET membrane surface. A number of apoptotic genes were downregulated in cells grown on the TCP surface after treating with the inhibitor, as compared to those expressed in cells grown on a TCP surface without inhibitor treatment (Figure 4D). These genes include BIRC2, CASP2, CASP7, DAXX, DIABLO, IKBKG, MAP3K5, MAPK8, and NGF. There was a 40% suppression in caspase-7, suggesting a successful inhibition on caspase-mediated apoptosis in cells grown on a TCP surface after inhibitor treatment. Most of the genes in Figure 4D were downregulated to levels similar to those in cells cultured on a PET membrane surface. Therefore, these experimental results demonstrated that a caspase-8 inhibitor efficiently prevents the apoptosis of hPSCs cultured on a TCP surface.

### 2.4. The Influence of Caspase-8-Mediated Pathway on the Metabolism of hPSCs Grown on the TCP Surface

**Figure 5:**
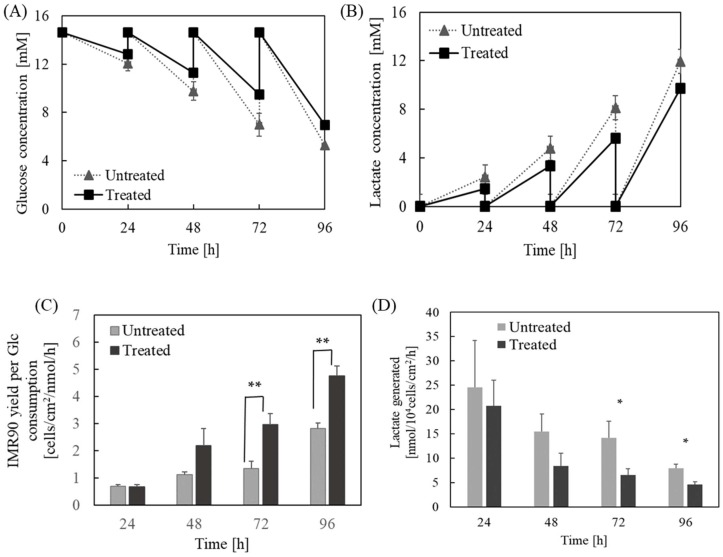
Suppression of caspase-mediated apoptotic pathway improved metabolism of iPSCs cultured on the TCP surface. iPSCs were treated with caspase-8 inhibitor (Treated) as described in Materials and Methods. Cells without the treatment (Untreated) served as a control. (**A**) The time course of glucose concentration and (**B**) lactate concentration in iPSC cultured on the TCP surface. (**C**) The time course of cell yield based on glucose (Glc) consumption. (**D**) The amount of lactate generated per glucose consumed. * *p* < 0.05; ** *p* < 0.01. Data shown were averages obtained from three independent experiments.

Furthermore, to examine whether the inhibition of caspase pathway also contributes to improved metabolism inside iPSCs grown on the TCP surface, we investigated the effect of caspase-8-mediated pathway on glucose consumption, lactate generation, as well as cell yield in cultures grown on TCP (Figure 5). Similar to the aforementioned, cells were incubated with caspase-8 inhibitor for 2 h post one day seeding. As expected, the inhibitor-treated cells were able to consume less glucose and generate less byproduct lactate, but maintain a relatively higher growth rate (Figure 4B and Figure 5A,B). The inhibitor treated cells possessed significantly higher yield per glucose consumed, particularly at day 3 and 4 (Figure 5C), and the yield of lactate generated decreased considerably (Figure 5D). Since lactate is generated by glycolysis, the reduced lactate level and increased cell yield per glucose consumed revealed an enhanced metabolic efficiency and favorable microenvironment for hPSCs self-renewal if caspase pathway was suppressed using TCP. 

## 3. Discussion

We have reported previously that the PET porous membrane substrate surface upregulates expressions of a number of extracellular matrix and cell adhesion molecules in hPSCs, leading to the activation of a β-catenin mediated *Wnt* signaling pathway and, consequently, augmented stem cell attachment and self-renewal. We also validated that hESCs remain undifferentiated when grown on PET membrane substrates after five consecutive passages [4]. In this study, we further scrutinized the physiological cues provided by the PET membrane for hPSC proliferation. We illustrated that the interaction of the PET membrane-hPSCs allows considerable depletion of caspase-mediated apoptosis, resulting in the enhancement of cell metabolism and proliferation. Importantly, the microenvironment of the traditional tissue culture plate for hPSC culture could be improved by a simple anti-caspase treatment to prevent cells from caspase-mediated apoptosis and further facilitate cell metabolism. The effect of a caspase-8 inhibitor on human iPSCs and hESCs has been investigated by other groups as well. The same inhibitor has been used to enhance the post-thaw survival rate of hESCs. The cells maintained their pluripotency after the inhibitor treatment [30,31]. 

FADD-DAXX-CASP8-CASP3/7 forms a direct apoptotic execution pathway. All the genes in the pathway were downregulated in the PET membrane culture, indicating that interaction between the PET membrane and hPSCs sends signaling to the cells for enhanced survival and attachment. DIABLO encodes a mitochondrial protein that enters the cytosol when cells undergo apoptosis, and activates caspases [32]. BIRC2 encodes Baculoviral IAP repeat containing 2 protein that inhibits apoptosis by binding to TNF receptor-associated factors (TRAFs), such as TRAF1 and TRAF2. Interestingly, both BIRC2 and TRAF2 showed more than 2-fold down-regulation in cells grown on the PET surface. This implies that the impaired caspase-mediated apoptosis resulted in the augmentation of the cell attachment and proliferation on the PET surface. Treating hPSCs grown on TCP surface with caspase-8 inhibitor resulted in the downregulation of BIRC2 and DIABLO, suggesting the two genes are negatively regulated by caspase-8 activity when taking their functions in the caspases pathway into consideration. In addition, after treatment with the caspase-8 inhibitor, the expression of caspase-2, MAP3K5, MAPK8, and DAXX were blocked to some degree, even though these genes are not downstream genes of caspase-8. Although MAP3K5 is involved in a c-Jun N-terminal kinase (JNK) pathway, it has close connections to the caspase pathway [33]. 

The results of our apoptotic signaling pathway analyses using PET and TCP surfaces are consistent with a previous study reporting that the PET membrane downregulates Rho-associated protein kinase (ROCK) and thus supports hESC self-renewal [26]. ROCK is activated by caspase cleavage, and its activity is directly associated with caspase-mediated apoptosis [34]. It plays pleiotropic roles in cell cytoskeletal reorganization, including actin formation, and thus directly influences cell attachment, proliferation, and gene expression [35,36]. Hence, ROCK inhibitor has been widely utilized in hPSC culture and differentiation in order to avoid apoptosis and enhance cell survival, proliferation, and differentiation [6,37]. Therefore, our experimental results coincide with previous work reported by others and our group [4,26]. These results also suggest that the PET membrane environment primarily suppresses apoptosis that may potentially induce overexpression of cytoskeleton genes. Furthermore, a recent study reported that lactate inhibits the growth of mouse embryonic stem cells (ESCs) and can even reduce the expression of pluripotent markers if lactate concentration is too high [38], which supports our finding that lactate production has negative impact on hPSC self-renewal. In our studies, since the lactate concertation is relatively low, hPSC pluripotency was not affected [4]. Taken together, our experimental results provided informative insights into signal pathways influencing hPSC proliferation and metabolism during routine maintenance and expansion of hPSCs on diverse surface matrices, which would shed light on large-scale preparation of stem cells for clinical applications.

## 4. Conclusions

In this study, we discovered that physicochemical cues of the PET membrane substantially improve hPSC metabolism by increasing the cell yield and inhibiting the generation of toxic byproduct. We investigated the mechanism underlying the improvement by the PET membrane platform. We found that the caspase-mediated apoptotic signaling pathway is significantly inhibited in cells cultured on a PET membrane surface. By blocking caspase-8-mediated apoptotic pathway in cells cultured on a traditional polystyrene tissue culture plate, we were able to mimic the microenvironment of the PET membrane using the tissue culture plate. Thus, the cellular apoptosis using a polystyrene tissue culture plate can be alleviated considerably by a quick and simple anti-apoptotic treatment.

## 5. Materials and Methods

### 5.1. Cell Culture and Monitor of Cell Proliferation

hESC line H9 and human iPSC line IMR90 acquired from the WiCell Research Institute (Madison, WI, USA) were routinely maintained in mTeSR1 medium (StemCell Technologies, Vancouver, BC, Canada) on growth factor reduced Matrigel-coated tissue culture dishes at 37 °C and 5% CO_2_ as described previously [4,5,39]. Cells were passaged by 1 mg/mL of dispase treatment and scraping every 3–4 days, with a split ratio of 1:3–1:6. The culture medium was exchanged daily. The morphology of cell colonies was examined daily, and spontaneously differentiated colonies were removed to ensure the maintenance of undifferentiated state of hPSCs. To characterize hESC and iPSC behavior under various conditions, ~5 × 10^4^ cells/cm^2^ were seeded to the PET porous membrane surface with 2 × 10^6^ pores/cm^2^ (EMD Millipore, Billerica, MA, USA) after Matrigel coating (Corning Inc., New York, NY, USA). Cells seeded onto Matrigel coated tissue culture polystyrene dishes, referred to as TCP, served as a control. Cell doubling time (*t*_d_) was calculated as follows:

(1) $$\frac{dx}{dt}=\mu x$$

(2) $$t_{d}=\frac{\ln2}{\mu }$$

where *x* is the cell concentration and *µ* is the specific growth rate. hPSC numbers were counted at 24-h intervals during the culture using a hemocytometer after trypan blue staining.

### 5.2. Measurement of Glucose and Lactate Concentrations

A glucose colorimetric assay kit II (Biovision Inc., Milpitas, CA, USA) was used to determine glucose concentration in hPSC cultures, according to the manufacturer’s instructions. A glucose standard curve was prepared in order to calculate glucose concentrations using a microplate reader Synergy H1 (BioTek, Winooski, VT, USA). Lactate produced by hPSCs was determined using a lactate colorimetric assay kit II from Biovision. A standard curve was prepared using a lactate standard solution, similar to the aforementioned glucose measurement. The lactate production per cell was estimated by dividing the amount of lactate generated by the cell number.

### 5.3. Quantitative Real Time-PCR

RNA was extracted from cells using EZNA HP total RNA kit from OMEGA (VWR international, Radnor, PA, USA). TaqMan array plates measuring signaling pathways were purchased from Applied Biosystems. The TaqMan RNA-to-Ct one-step reagent from Applied Biosystems was used to determine gene expression using a CFX Connect System from Bio-Rad, as described elsewhere [4,5,39]. Using the Ct value of all the genes in the array, the fold change was calculated through the ∆∆*C*t algorithm. GAPDH was used as a housekeeping gene.

### 5.4. Caspase-8 Inhibitor Z-IETD-FMK Treated Cell Culture

Caspase-8 inhibitor, Z-IETD-FMK, was purchased from Selleckchem (Houston, TX, USA). The caspase inhibitor was added to the medium at 24 h post seeding. After 2 h of incubation, the inhibitor was removed from the culture medium, and the cell culture continued for days as indicated.

### 5.5. Statistical Analysis

All experiments were carried out in at least triplicate. The means and standard deviations of experimental results were computed and a *p*-value of 0.05 or less was considered a statistically significant difference between two groups of experiments.
